# Network Flow Method Integrates Skeleton Information for Multiple *C. elegans* Tracking

**DOI:** 10.3390/s25030603

**Published:** 2025-01-21

**Authors:** Taoyuan Yu, Xiping Xu, Ning Zhang

**Affiliations:** School of Optoelectronic Engineering, Changchun University of Science and Technology, 7089 Weixing Road, Changchun 130022, China; 2019100228@mails.cust.edu.cn (T.Y.); zhangning@cust.edu.cn (N.Z.)

**Keywords:** multiple *C. elegans* tracking, network flow method, skeleton algorithm, adjacent frame match

## Abstract

In order to solve the issues arising from collisions, this paper proposes a network flow method combined with skeleton information for multiple *C. elegans* tracking. In the intra-track stage, non-colliding *C. elegans* are identified and associated as trajectory fragments based on their motion and positional information, and colliding *C. elegans* are then segmented based on an improved skeleton algorithm and matched as trajectory fragments. Subsequently, the trajectory fragments are employed as vertices to construct a network flow model. The minimum-cost method is then utilized to solve the model, thereby obtaining the optimal solution for the multiple *C. elegans* trajectories. The proposed method was evaluated using video data of the *C. elegans* population at three distinct ages: L4, young adult, and D1. The experimental results demonstrate that the method proposed in this paper exhibits a MOTA between 0.86 and 0.92, and an MOTP between 0.78 and 0.83, which indicates that the proposed method can be employed in multiple *C. elegans* tracking. It is our hope that this method will prove beneficial to *C. elegans* laboratories, offering a novel approach to multiple *C. elegans* tracking.

## 1. Introduction

The *Caenorhabditis elegans* (*C. elegans*) has been employed as a model organism in neuroscience, genetics, and others research fields [1,2,3,4,5]. A substantial body of research has demonstrated the significant research value of accurately acquiring the trajectory of worm in behavior analyses [6,7,8,9]. The locomotive behavior of *C. elegans*, as an expression of the interconnection and collaboration between neurons, can reveal basic neural mechanisms and provide important clues in understanding motor control in complex animals [2]. By observing their movement patterns, researchers can explore their responses to environmental stimuli, such as food, drugs, and pollutants [1,5]. The trajectory of movement is the most intuitive representation of locomotive behavior, which is becoming increasingly in demand [7]. The multi-worm tracking method has attracted the attention of more researchers, which is used to extract the trajectories of all worms in the field of view [8].

Multi-worm tracking methods proposed in recent years have been widely used in practice. For example, the real-time Multi-Worm Tracker (MWT) and Multi-Animal Tracker (MAT) have been proposed for the acquisition of population trajectory to analyze properties such as chemotaxis and tap habituation [7,8]. The main idea is to detect the worm responses within each image frame through the image segmentation techniques, subsequently linking these responses to construct a trajectory. However, due to the high similarity of individual worms, the tracking errors caused by collision have become one of the biggest challenges in multi-nematode tracking. This results in a new ID being assigned to the worm after a collision, and the tracking trajectory is incoherent [7]. Additionally, several deep-learning-based multi-worm tracking methods have been proposed. These include the introduction of Faster R-CNN and YOLOv5 for the purpose of behavior and motility analysis [6,10]. The main idea of these methods is to enhance the precision of worm detection through prior learning. However, the preliminary work of the learning-based approach may necessitate the labeling of thousands of worm images, which is a labor-intensive process [6,8,10].

From a macroscopic perspective, multi-object tracking can be classified into two steps: the intra-trajectory stage and inter-trajectory stage [11]. The aforementioned methods are primarily concerned with the intra-trajectory stage. Peter et al. designed a global optimization framework based on the inter-trajectory method for enhancing the precision of multi-worm tracking that utilizes the results of MWT as inputs [9]. This framework merges trajectory segments into the trajectory by unraveling collisions and pruning methods. Nevertheless, this framework necessitates the deduction of colliding worm positions and the pruning of trajectories during the inter-trajectory processing stage, which inevitably increases the processing time and uncertainty. In order to achieve more accurate multi-worm tracking, in the event of worm collision, we hope to enhance both the intra-trajectory and inter-trajectory stages, drawing inspiration from the aforementioned approach.

In this paper, we propose a multi-worm tracking method that combines network flow methods and skeleton information. First, in the intra-trajectory stage, the worm responses in all frames of the video are obtained by a detector as well as the determination of whether a worm collision occurs or not. In the event of a worm collision, the colliding trajectory is obtained through the improved skeleton method. Conversely, the worm response is associated with a trajectory fragment based on the spatio-temporal features. This indicates that the worm collision problem can be solved within the intra-trajectory stage, thereby enhancing tracking precision. Subsequently, in the inter-trajectory stage, the worm trajectory fragments are employed as vertices to construct a network flow model. The worm motion features are then utilized as a cost function to solve the network flow based on the minimum cost to obtain the multi-worm trajectory. It is important to note that this method employs the trajectory fragments as the vertices of the network flow model. Each vertex is capable of containing both static and dynamic features, which enhances the precision of data association while simultaneously reducing the computational time. Experiments were carried out on worm populations videos at different ages, and the results showed that the proposed method can accurately track freely moving worm populations.

## 2. Materials and Methods

### 2.1. Worm Video Acquisition

The camera utilized in the experiment has a resolution of 5120 × 5120, a pixel size of 2.5 × 2.5 microns, and is capable of capturing four frames of images per second. An industrial lens with a focal length of 35 mm was selected and connected to the camera via a C-mount. An image with the dimensions of 60 × 60 mm can be captured when the work distance is set to 235 mm. A plane light source with an area of 65 × 65 mm was employed as the illumination source, illuminated by backlighting [12]. Under this experimental setup, a worm with a length of 1 mm and a width of 0.15 mm was represented by 85 × 12 pixels. The strain of worm used in the experiment was N2 wild type. The worm eggs were placed in a 30 mm petri dish coated with *Escherichia coli* OP50, and the researchers recorded videos of the worms at three times: 48, 60, and 72 h.

### 2.2. Multi-Worm Tracking Method

**Figure 1:**
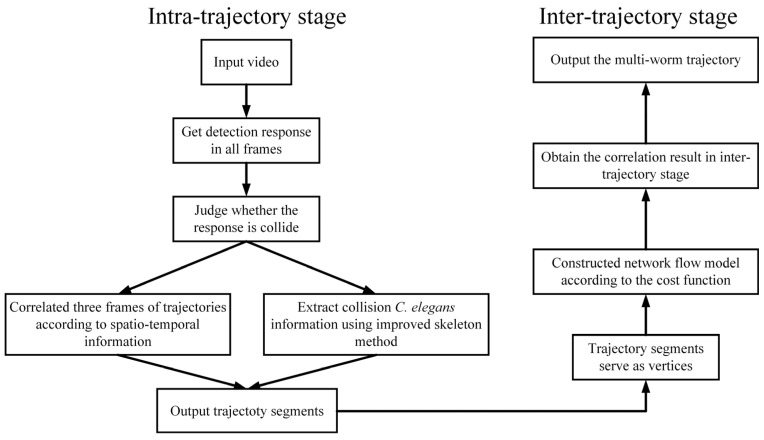
Flowchart of the proposed multi-worm tracking method.

The proposed method for tracking worms is illustrated in Figure 1, and it can be divided into the intra-trajectory stage and inter-trajectory stage. In the intra-trajectory stage, the responses of the worm in all frames are initially obtained, and the responses of the worm that are occluded are extracted separately. The processing of colliding and non-colliding worms employs different strategies, and the response results of every three frames are correlated to obtain trajectory fragments. Subsequently, in the inter-trajectory stage, the trajectory fragments are used as vertices to construct a network flow model, which is solved to obtain the multi-worm trajectory.

### 2.3. Worm Detection and Colliding Judgment

**Figure 2:**
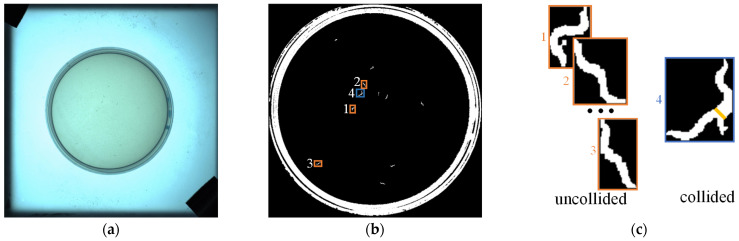
Flowchart of worm detection and judgement. (**a**) Original image. (**b**) Binary image of petri dish. (**c**) Worm collision judgement. Four representative blobs, labeled 1, 2, 3, and 4, were selected in (**b**). The corresponding enlarged views are shown in (**c**).

Figure 2 illustrates the detection and colliding worm judgment within the video frame. Figure 2a depicts the original video frame, and a morphological method is employed to derive a binary image of the worm population [13], as illustrated in Figure 2b. At this point, the blob in Figure 2b is the detected worm response, and the centroid and skeleton of the worm can be extracted. In order to classify the worm responses into collided and uncollided responses, the area threshold $T_{area}$ is introduced. When the area of a worm response is greater than $T_{area}$, it is classified as a collided response; otherwise, it is classified as an uncollided response. Four blobs are selected for demonstrating the judge method in Figure 2b, as shown in the four rectangles in the figure, where the orange rectangle is the uncollided worm response, and the blue rectangle is the collided worm response. The magnified view is shown in Figure 2c, where the yellow area in rectangle 4 is the collision area of the worms. In this paper, the area of the worm individual is about 150–250 pixels, so the $T_{area}$ is set to 300. It is worth noting that different values of $T_{area}$ should be chosen when applied to different datasets.

### 2.4. Matching Method of Non-Colliding Worm

**Figure 3:**
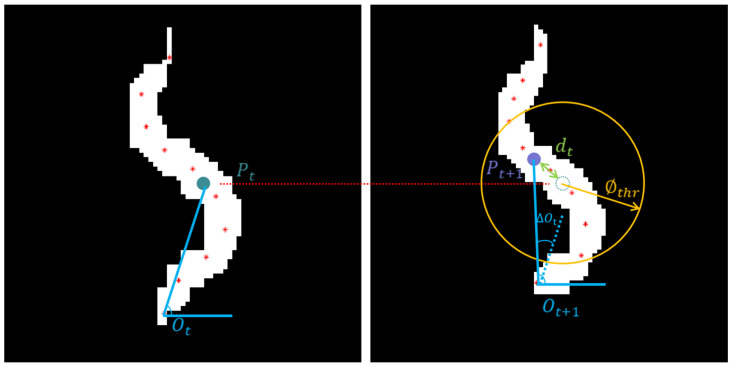
Image of the matching method for the center points of the skeletons in adjacent frames. Wherein, the red asterisk is skeleton point, the solid green and purple circles in the left and right images are the center point of the skeleton, the dotted green circle in the right image is the center point of the skeleton at the previous time, and the yellow circle in the right image is the motion range of worm.

In order to obtain the trajectory fragments of the non-colliding worm, the center skeleton points p(x,y) of the adjacent frames are matched according to the temporal and spatial characteristics of the worm [14]. Figure 3 illustrates the binary images of the worm in frames t and t+1, as shown in the left image and the right image. Wherein, $P_{t}$ and $P_{t+1}$ represent the center points of the skeleton, $d_{t}$ denotes the Euclidean distance between $P_{t+1}$ and $P_{t}$, and $\emptyset_{thr}$ represents the distance threshold. The movement direction of the worm is represented by the angle formed by the skeleton center point and the skeleton short point with the horizontal direction, which are $O_{t}$ and $O_{t+1}$, respectively. The absolute value of the difference between $O_{t}$ and $O_{t+1}$ is expressed as $∆O_{t}$, and the angle threshold is indicated by $O_{thr}$. In order to determine the matching of $P_{t}$ and $P_{t+1}$ in adjacent frames, the following formula needs to be satisfied:

(1) $$f_{match}=\left\{\begin{array}{c} 1,\;{d_{t}\leq \emptyset }_{thr}\;\cap \;∆O_{t}\leq O_{thr}\\ 0,\;else \end{array}\right.$$

### 2.5. Tracking Method of Colliding Worm with Improved Skeleton Method

**Figure 4:**
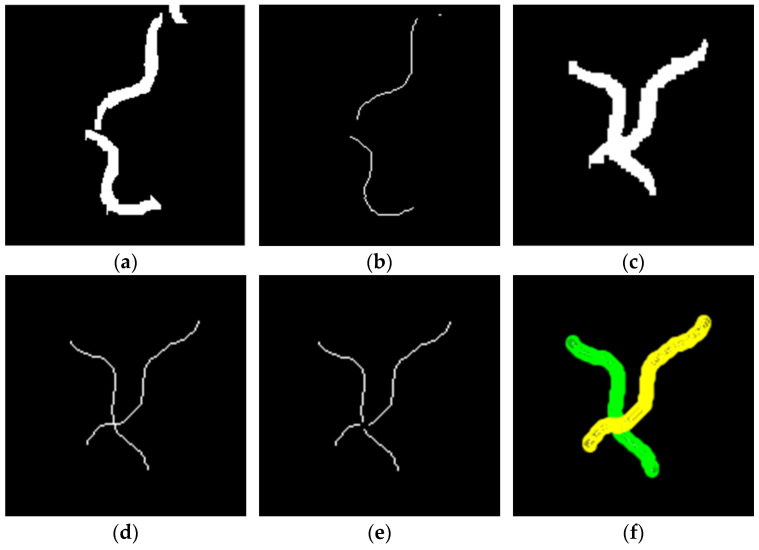
Flow chart of improved skeleton algorithm. (**a**) The worm blob before collision occurs. (**b**) The traditional skeleton method result of (**a**). (**c**) The worm blob when collision occurs. (**d**) The traditional skeleton method result of (**c**). (**e**) The skeleton without branchpoint. (**f**) The improved skeleton method result of (**c**). The yellow and green mask in (**f**) represent skeleton of different worms.

In contrast to non-colliding worms, the data of colliding worms cannot be directly correlated with trajectory fragments. In this paper, an improved skeleton method is employed for the segmentation of worms, as illustrated in Figure 4. Among them, Figure 4a shows the outlines of the two worms before the collision, and their skeleton information can be obtained using the traditional skeleton method, as shown in Figure 4b [15,16]. Subsequently, the worm collisions occur, as shown in Figure 4c. It can be seen that using the traditional skeleton method can only obtain a single skeleton, and the exact position of the colliding worms cannot be determined, as shown in Figure 4d. To solve this problem, the branchpoint of the traditional skeletons is removed, as shown in Figure 4e. At this point, there are four skeletons in the figure. Based on the skeleton length information recorded in Figure 4b, the most suitable skeleton combination is found using the dynamic matching method, as shown in Figure 4f [17]. Wherein, the green and yellow markers in the figure correspond to the separated worm skeletons. Comparing Figure 4d,f, it can be seen that the improved skeleton method effectively distinguishes the position of the collided worms.

**Figure 5:**
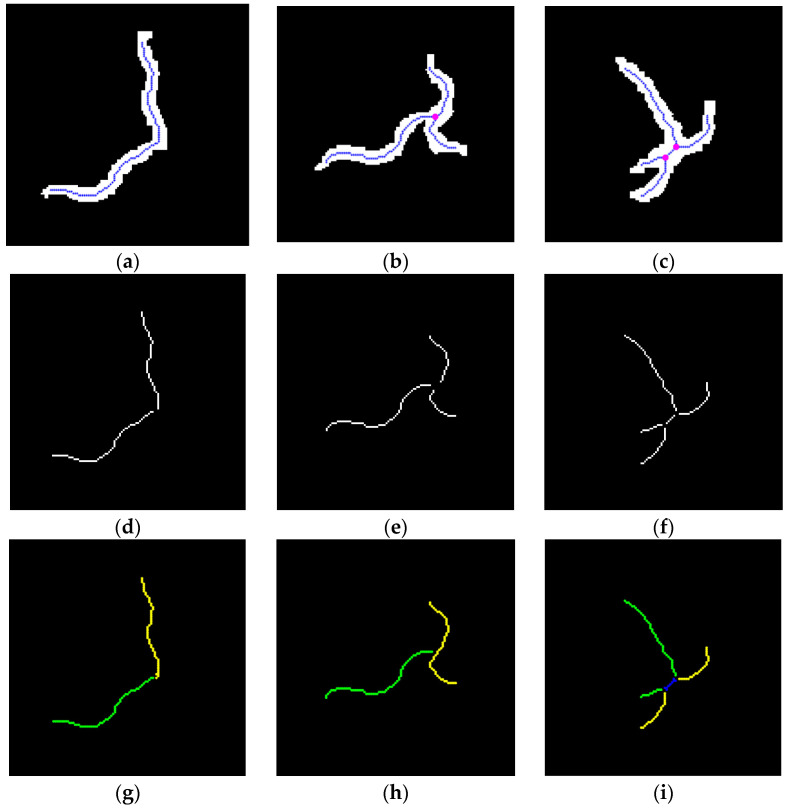
Flowchart image of improved skeleton algorithm for colliding worms. (**a**–**c**) Worm blob and skeleton in different situations. (**d**–**f**) The skeleton diagram without branchpoints. (**g**–**i**) The result image of improved skeleton algorithm. The blue line in (**a**–**c**) is the traditional skeleton, the magenta circles in (**a**–**c**) are the branchpoints, the green and yellow line in (**g**–**i**) is the skeleton of collision worm, and the blue line in (**i**) is the skeleton shared by two worms.

Subsequently, we applied the improved skeleton method to different worm collision situations, as shown in Figure 5a–c. The number of skeletons branchpoints is 0, 1, and 2, respectively. In Figure 5a–c, the white area is the worm blob, the blue curve is the result of the traditional skeleton algorithm, and the red dots are the branchpoints of the skeleton. After removing the branchpoints on the skeleton, as shown in Figure 5d–f, the skeleton in the figure is assigned using the dynamic matching method to obtain the most reasonable skeleton result, as shown in Figure 5g–i. Among them, green and yellow represent the skeletons of the two worms, and the blue line represents the area shared by the two worms. Once all the colliding worm fragments have been split, the method proposed in Section 2.4 should be employed to obtain the trajectory fragments.

### 2.6. Network Flow Model

The trajectory fragments generated by the colliding and non-colliding worms are represented by $X_{i}\left(x,\;y,\;v,\;a,\;t\right),\;X_{i}\in X$, which is the vertex of the network flow model [14]. Wherein, (x, y) is the position, v is the velocity, a is the appearance, and t is the current frame index. The trajectory of the *k*th worm is defined as $T_{k}=\{X_{k1},X_{k2},\ldots ,X_{kn}\}$, and the set of all trajectories is defined as T [18]. The maximum a posteriori probability of T can be defined by Formula (2):

(2) $$T^{*}={argmax}_{T}P\left(T|X\right)\;={argmax}_{T}P\left(X|T\right)P\left(T\right)\;={argmax}_{T}\prod_{i}P\left(X_{i}|T\right)P\left(T\right)$$

Assuming that the vertices are independent, Formula (2) can be transformed into Formula (3), as follows:

(3) $$T^{*}={argmax}_{T}\prod_{i}P\left(X_{i}|T\right)\prod_{T_{k}\in T}P\left(T_{k}\right)$$

The first term in Formula (3) represents the likelihood of observing $X_{i}$, and the second term is a Markov chain that can be expressed as follows:

(4) $$P\left(T_{k}\right)=P_{enter}\left(X_{k1}\right)P_{link}\left(X_{k2}|X_{k1}\right)P_{link}\left(X_{k3}|X_{k2}\right)\ldots P_{link}\left(X_{kn}|X_{kn-1}\right)P_{exit}\left(X_{kn}\right)$$

Among them, $P_{enter}$, $P_{link}$, and $P_{exit}$ are the birth probability, transition probability, and death probability of the vertices, respectively. Since each vertex can only be used once, the Bernoulli distributions $f_{en,i}$, $f_{ex,i}$, $f_{i,j}$, and $f_{i}$ are introduced for each term in Formula (4). In this way, Formula (3) can be transformed into Formula (5), as follows:

(5) $$T^{*}={argmin}_{T}\sum_{T_{k}\in T}-logP\left(T_{k}\right)+\sum_{i}-logP\left(X_{i}|T\right)\;={argmin}_{T}\sum_{i}C_{en,i}f_{en,i}+\sum_{i}C_{ij,}f_{i,j}+\sum_{i}C_{ex,i}f_{ex,i}+\sum_{i}C_{i}f_{i}$$

where the formulas of $f_{en,i}$, $f_{ex,i}$, $f_{i,j}$, and $f_{i}$ are as follows:

(6) $$f_{en,i}=\left\{\begin{array}{c} 1\;\;\;T_{k}\;starts\;from\;X_{i}\\ 0\;esle \end{array}\right.$$

(7) $$f_{ex,i}=\left\{\begin{array}{c} 1\;\;\;T_{k}\;ends\;at\;X_{i}\\ 0\;esle \end{array}\right.$$

(8) $$f_{i,j}=\left\{\begin{array}{c} 1\;\;\;X_{j}\;follows\;X_{i}\\ 0\;esle \end{array}\right.$$

(9) $$f_{i}=\left\{\begin{array}{c} 1\;\;\;T_{k}\;\in \;X_{i}\\ 0\;esle \end{array}\right.$$

**Figure 6:**
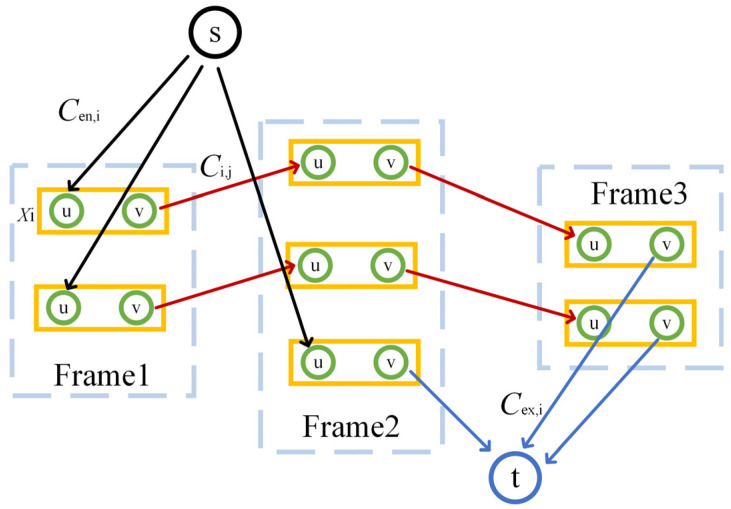
The structure diagram of network flow model.

The schematic structure of the network flow model is shown in Figure 6, where $X_{i}$ is regarded as the vertex, as shown by the yellow rectangle, and s and t are the source and sink points, as shown by black and blue circles. The pair of nodes is internally connected by $C_{i}$ with a flow of $f_{i}$; the node and source are connected by $C_{en,i}$ with a flow of $f_{en,i}$, as shown by the black arrow; the node and sink point are connected by $C_{ex,i}$ with a flow of $f_{ex,i}$, as shown by red arrow; and different nodes are connected by $C_{i,j}$ with a flow of $f_{i,j}$, as shown by the blue arrow. The solution of the network flow model can be obtained by connecting the nodes between adjacent frames; therefore, the minimum cost flow method was introduced to find the optimal solution [14].

The cost function $C_{i,j}$ of adjacent vertices based on the position and velocity information of a worm can be expressed as follows:

(10) $$C_{i,j}=\omega_{a}C_{a}+\omega_{v}C_{v}$$

where $C_{a}$ and $C_{v}$ represent the appearance cost and velocity cost, respectively. $\omega_{a}$ and $\omega_{v}$ represent the corresponding weights, which are set to 0.5 in this paper. The appearance cost $C_{a}$ is obtained from the grayscale value of the worm. The skeleton points of the worm in Figure 3 are used as the center to generate an M × M mask, and the average grayscale value is calculated by the following formula [19]:

(11) $$\mu_{i}=\frac{1}{M^{2}}\sum_{I=1}^{M}\sum_{J=1}^{M}P(I,J)$$

The Bhattacharyya distance is used to calculate the similarity of the appearance q and c between two worms as $C_{a}$, as shown in Formula (12) [20]:

(12) $$D\left(q,c\right)=\sqrt{1-\sum_{l=1}^{L}\sqrt{q_{l}c_{l}}}$$

where L represents the number of skeleton points, which is 10 in this paper. The velocity cost is calculated based on the cosine similarity, as shown in Formula (13):

(13) $$V\left(v_{1},v_{2}\right)=\frac{v_{1}\cdot v_{2}}{\left\|v_{1}\right\|\cdot \left\|v_{2}\right\|}$$

wherein, $v_{1}$ and $v_{2}$ represent the velocity of two vertices.

## 3. Experiment Results and Analysis

### 3.1. Multi-Worm Tracking Result

**Figure 7:**
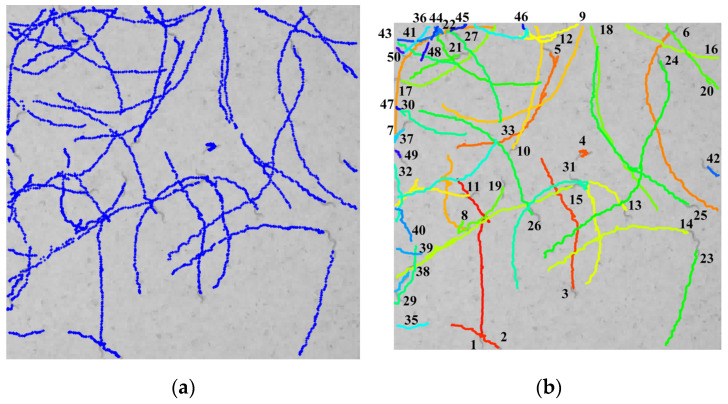
Experimental results of proposed multi-worm tracking method. (**a**). Output trajectory fragments of the intra-trajectory stage. (**b**) Output multi-worm trajectory of the inter-trajectory stage. The blue point in (**a**) is all the responses that were detected, the line of different color in (**b**) is different trajectories, and the number 1–50 is the ID of worms.

The multi-worm tracking experiment was conducted on the video of the L4 stage worm. A video comprising 1200 frames and a resolution of 1080 × 1080 was extracted from the original video, which had a resolution of 5120 × 5120, and the results of the tracking are illustrated in Figure 7. In Figure 7a, the blue points represent the trajectories of all the worms, while Figure 7b depicts the output of the multi-worm tracking method, wherein the curves of varying colors correspond to the trajectories of different worms. The experimental results demonstrate that a total of 50 trajectories were successfully identified from the worm responses, and the collision trajectories can also be distinguished.

### 3.2. Quantitative Analysis of C. elegans at Different Ages

**Figure 8:**
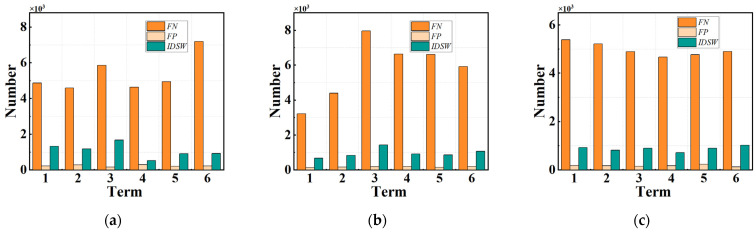

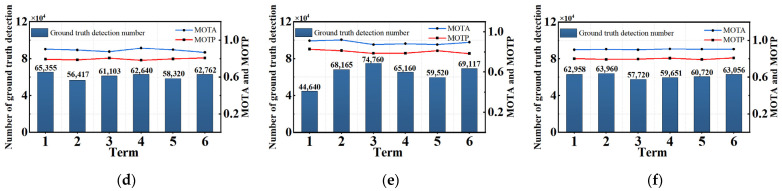
The diagram of the experiment result of the proposed method. (**a**–**c**) The *FN*, *FP*, and *IDSW* for the method at different ages. (**d**–**f**) The *GT*, MOTA, and MOTP for the method at different ages.

In order to evaluate the performance of the multi-worm tracking method proposed in this paper, the typical MOT evaluation criteria are introduced, including multiple-object tracking accuracy (MOTA), multiple-object tracking precision (MOTP), and ID switch (IDSW) [21]. Among them, the definition of MOTA is shown in Equation (14):

(14) $$MOTA=1-\frac{FN+FP+IDSW}{GT}$$

where FN and FP are the numbers of missed detection and false detection, GT is the number of true detections, and IDSW is the number of ID switches. The MOTP is defined as shown in Equation (15):

(15) $$MOTP=\frac{\sum_{t,\bar{i}}d_{t,\bar{i}}}{\sum_{t}c_{t}}$$

where $d_{t,\bar{i}}$ is the intersection ratio of the algorithm detection box and the truth detection box, t is the number of frames, $\bar{i}$ is the algorithm check box, and $c_{t}$ is the total number of correct matches in the tth frame. A higher MOTA and MOTP indicate superior algorithm performance. Experiments were conducted on videos of the worm population at three stages: L4, young adult, and D1, and each stage consisted of six videos of 1200 frames. The experimental results are shown in Figure 8, where Figure 8a–c represent the FN, FP, and IDSW of the L4, young adult, and D1 stages, and the ground truth detection number is shown in Figure 8d–f. It can be observed that the primary source of error in the algorithm originates from FN, which is attributable to the fact that the worm detection algorithm employed is an adaptive threshold method, which can result in the deletion of minor worm blobs. In comparison to FN, the proportion of FP is significantly lower. This is primarily attributed to the presence of impurities in the background and the inadvertent recognition of worm parts at the edge of the image as targets. Additionally, some errors in the algorithm are attributed to IDSW, resulting from the misidentification of worms in the same direction that are in close proximity. Figure 8d–f illustrate the GT, MOTA, and MOTP of the L4, young adult, and D1 stages, respectively. It can be seen that the MOTA of the algorithm is between 0.86 and 0.92, which is caused by the FN, FP, and IDSW mentioned above. Furthermore, the figure illustrates that the MOTP of the algorithm ranges between 0.78 and 0.83. This is attributed to the fact that the contour of the worm blob identified by the worm detector was smaller than the truth contour of the worm, resulting in the value of $d_{t,\bar{i}}$ being less than one.

### 3.3. Comparative Experiment

To further demonstrate the practicality of the proposed method, we conducted a comparative experiment with the deep-worm tracker reported in 2023 [10]. The dataset comprises the four different worm videos provided in the deep-worm tracker. The experimental results are shown in Table 1. Video Worm1 contains one worm, which moves out of the field of view at 30 s. The MOTA of the proposed method is 97.3%, and the main error comes from the worm detection method based on the morphological algorithms. When part of the worm body moves out of the field of view, the area of the worm blob is less than the threshold $T_{area}$, and the detector makes an error, resulting in an increase in FN. Video Worm2 contains about 40 worms, some of which are larvae and some of which are adults. The MOTA of the proposed method is about 86%. Since the difference between the bodies of larva worms and adult worms is large, the detector only detects adult worms, which leads to a large FN error. Video Worm3 contains ten worms, of which four worms move out of the field of view. The MOTA of the proposed method is about 91%, and the error mainly comes from FN caused by worm movement out of the field of view and IDSW caused by worm collisions. Video Worm4 contains 11 worms. The MOTA of the proposed method is about 91%, which is due to the movement of a worm at the edge of the video from 0 to 25 s, which causes some IDSW. The experimental results show that the proposed method maintains good stability in different situations.

## 4. Discussion

In this paper, we propose a multi-worm tracking method that combines the network flow method with skeleton information. It is our contention that this method represents an effective approach to offline multi-worm tracking. The proposed method can obtain multi-worm trajectories without the necessity of a pre-learning process, as shown in Figure 7. In addition, this paper proposes an improved skeleton method for collision worm skeleton segmentation. As can be seen in Figure 4d and Figure 5a–c, the skeleton obtained by the traditional skeleton method cannot distinguish the collision worm skeleton information. Based on the traditional skeleton method, we introduced the skeleton branchpoint information and blob information for improvement. As can be seen in Figure 4f and Figure 5g–i, the improved skeleton method can segment the collision worm skeletons. This method is simple and effective and can solve the shortcomings of the traditional skeleton method in handling collision situations.

However, there are still some shortcomings in the proposed method. As shown in the experimental results in Figure 8d, *FN*, *FP*, and *IDSW* still occur to some extent. Among them, the main source of error comes from the morphological-method-based worm detection method. When there are worms of different sizes in the field of view, the detection method can only extract the responses of worms of similar sizes. Some of the errors come from the network flow method of the inter-trajectory stage. This paper uses position, appearance, and motion speed as worm features. When there are collisions that cannot be judged by appearance and speed, the network flow method will make a judgment error. Therefore, we believe that the main improvement methods are as follows: First, a more advanced worm detection method can be used. This idea can be achieved by combining the optimization theory with a probability-based detector to find the pixels in the image that belong to the worm, so as to obtain a more accurate worm response, which can eliminate the impact of differences in worm size. Alternatively, a learning-based detector can be used to extract worm detections in each frame, but this method requires a separate workstation to complete. Second, more worm features can be combined as the cost function of the network flow model; when the responses of approaching worms cannot be distinguished by appearance and speed, the motion features of the worms, such as amplitude and frequency of movement, can also be used as important features to improve tracking accuracy. In future works, we will optimize the above two points to further enhance the performance of the proposed method.

This paper proposes a multi-worm tracking method that integrates skeleton information and the network flow approach. The proposed method improves the accuracy of multi-worm tracking by optimizing both the intra-trajectory and inter-trajectory methods. Furthermore, this paper demonstrates the potential to achieve enhanced multi-worm tracking accuracy. It is hypothesized that this method can serve as an effective offline multi-worm tracking method and can be widely adopted in *C. elegans* laboratories, particularly in scenarios involving numerous worm collisions. By recording and analyzing the behavior of worms of different strains, researchers can better understand the relationship between the nervous system and motor behavior. In addition, the method proposed in this paper can be modified and applied to other microscopic organisms or medical images, which will play a role in the field of biological data analysis. In conclusion, the proposed method offers researchers a new idea to improve multi-worm tracking algorithms, extending beyond the scope of intra-trajectory or inter-trajectory methods.

## 5. Conclusions

In order to solve the tracking error caused by collisions in the multi-worm tracking method, this paper proposes a network flow tracking method that combines skeleton information with multi-worm tracking. First, morphological algorithms are used to obtain the responses of all worms in the video frame. For colliding worms, this paper uses an improved skeleton method to segment the position of the worms. Then, a spatial-information-based adjacent frame matching method is used to associate the responses of colliding and non-colliding worms in every three frames as trajectory fragments, which not only improves the accuracy of tracking but also reduces the time of the subsequent processing. Finally, the trajectory fragments are regarded as vertices to construct a network flow model, and a cost calculation method combining appearance features and motion features is used to solve the network flow model to obtain the optimal solution of the multi-worm trajectory. The proposed method is applied to the worm videos at different ages. The experimental results show that the MOTA of the method is between 0.86 and 0.92, which means the trajectories of multiple worms can be accurately obtained. The method proposed in this paper offers novel insights into the field of multi-worm tracking, facilitating a more comprehensive understanding of worm movement behavior research. The method presented in this paper introduces novel concepts in the field of multi-worm tracking and can be readily implemented in *C. elegans* laboratories.

## Figures and Tables

**Table 1:** The experimental result of the four worm videos.

Video File	Number of Worms	Duration of Video	MOTA	
			Deep-Worm Tracker [10]	Proposed Method
Worm1	1	0–30 s	99.60%	97.30%
Worm2	40	0–10 s	70.44%	86.60%
	44	10–20 s	63.16%	86.50%
Worm3	9	0–10 s	85.66%	91.20%
	9–10	10–20 s	70.88%	91.70%
	10	20–30 s	47.70%	90.60%
Worm4	11	0–10 s	82.18%	90.50%
	11	10–20 s	69.63%	91.30%
	11	20–30 s	34.90%	90.80%

## Data Availability

The original contributions presented in this study are included in the article. Further inquiries can be directed to the corresponding author.
